# FDG PET Imaging of the Pain Matrix in Neuropathic Pain Model Rats

**DOI:** 10.3390/biomedicines11010063

**Published:** 2022-12-27

**Authors:** Yilong Cui, Hiroyuki Neyama, Di Hu, Tianliang Huang, Emi Hayashinaka, Yasuhiro Wada, Yasuyoshi Watanabe

**Affiliations:** 1 Laboratory for Biofunction Dynamics Imaging, RIKEN Center for Biosystems Dynamics Research, Kobe 650-0047, Hyogo, Japan; 2 Laboratory for Pathophysiological and Health Science, RIKEN Center for Biosystems Dynamics Research, Kobe 650-0047, Hyogo, Japan

**Keywords:** neuroimaging, FDG, neuropathic pain, objective biomarker, pain matrix, preclinic

## Abstract

Pain is an unpleasant subjective experience that is usually modified by complex multidimensional neuropsychological processes. Increasing numbers of neuroimaging studies in humans have characterized the hierarchical brain areas forming a pain matrix, which is involved in the different dimensions of pain components. Although mechanistic investigations have been performed extensively in rodents, the homologous brain regions involved in the multidimensional pain components have not been fully understood in the rodent brain. Herein, we successfully identified several brain regions activated in response to mechanical allodynia in neuropathic pain rat models using an alternative neuroimaging method based on 2-deoxy-2-[^18^F]fluoro-d-glucose positron emission tomography (FDG PET) scanning. Regions such as the medial prefrontal cortex, primary somatosensory cortex hindlimb region, and the centrolateral thalamic nucleus were identified. Moreover, brain activity in these regions was positively correlated with mechanical allodynia-related behavioral changes. These results suggest that FDG PET imaging in neuropathic pain model rats enables the evaluation of regional brain activity encoding the multidimensional pain aspect. It could thus be a fascinating tool to bridge the gap between preclinical and clinical investigations.

## 1. Introduction

Pain is an unpleasant subjective experience that interacts with multidimensional neuropsychological processes, including sensory discrimination, cognitive evaluation, and emotional affection [1]. Chronic pain is the most common cause for seeking medical care, affecting over 20% of adults worldwide [2]. Unfortunately, most patients with chronic pain are not satisfied with currently available analgesic therapy [3], suggesting that the development of more effective therapies for chronic pain is indispensable. Pain is a multidimensional neuropsychological process and is not linearly related to primary nociception, especially in chronic pain. Owing to such a subjective nature and complex interactions with conscious brain activity, non-invasive neuroimaging has received increasing attention as a potential biomarker for the objective assessment of pain and the comprehensive exploration of pharmacological targets of pain intervention [4,5,6,7]. Previously, neuroimaging studies in patients have attributed structural abnormalities and functional alterations to chronic pain [7,8,9,10]. In patients with chronic back pain, the gray matter density was decreased in the prefrontal cortex and thalamic region [11], whereas the functional connectivity between the prefrontal cortex and the nucleus accumbens was increased [9]. Functional neuroimaging studies have also characterized several regions of the brain that are thought to be involved in different dimensions of pain components. The lateral thalamus, sensory cortex, and posterior insular cortex are preferentially related to the sensory-discriminative dimension of pain [5,7,12]. The medial thalamus and anterior cingulate cortex seem to be associated with the emotional affective dimension of pain [1,13,14], whereas the prefrontal cortex is related to the cognitive evaluation dimension of pain [15,16].

Meanwhile, preclinical research employing diverse animal models that mimic certain forms of clinical pain has been extensively undertaken to explore the pathophysiology of pain and to identify effective therapeutic targets for pain treatment [17,18,19,20]. However, due to the lack of reliable coherent biomarkers for pain assessment throughout preclinical and clinical studies, the pathophysiology revealed in these preclinical studies has not been completely translated into clinical practice [21]. In general, reflex-based behavioral observations are often used for pain assessment in most preclinical studies [22]. However, self-reporting-based subjective evaluation has long been used as the gold standard in clinical endpoints [3,23,24]. Such inconsistencies between the biomarkers of pain assessment in preclinical and clinical research may also hinder our understanding of the pathophysiology of chronic pain.

Current advances in neuroimaging technologies provide a potential consistent biomarker for pain assessment throughout preclinical and clinical research. A growing number of neuroimaging studies, involving methods such as functional magnetic resonance imaging (fMRI) and positron emission tomography (PET), have been performed in rodent pain models and have highlighted that functional and structural changes in several brain regions may underlie the pathophysiology of chronic pain [25,26]. However, most neuroimaging studies in rodents require the immobilization of the animal with anesthesia or a specific head-fix system, which may interfere with normal neuropsychological processes and cause a reduction of neuronal activity [25,27,28]. Compared with the conscious condition, the cerebral glucose metabolic rate in the cerebral cortex of mice was decreased by 66% under isoflurane, one of the most frequently used anesthetics in animal neuroimaging studies [28]. Recently, an alternative 2-deoxy-2-[^18^F]fluoro-d-glucose (FDG) PET imaging, which does not require immobilization, has been increasingly used for analyzing brain activity in rodents [29,30,31,32]. FDG is taken up by active regions of the brain and remains within the regions for at least an hour [33]; therefore, brain activity free from immobilization can be obtained by subsequent FDG PET scans performed under anesthesia, in which most FDG is taken up under a conscious condition prior to the PET scan. Using the FDG PET imaging and subsequent voxel-based statistical analysis, we investigated chronic pain-related brain activity in the spinal nerve ligated (SNL) neuropathic pain rat model.

## 2. Materials and Methods

All experimental protocols of our study were approved by the Animal Care and Use Committee of RIKEN, Kobe Branch, and were performed in accordance with the Principles of Laboratory Animal Care (NIH publication No. 85–23, revised 2011). This study was conducted in accordance with the Animal Research: Reporting in Vivo Experiments (ARRIVE) guidelines. All recommended means were followed to minimize animal suffering.

### 2.1. Neuropathic Pain Animal Model Preparation

**Figure 1:**
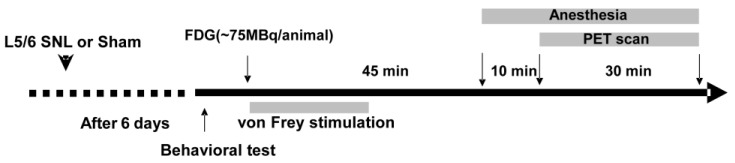
Experimental paradigm. The left L5 and L6 spinal nerve ligation (SNL) was performed one week before the FDG PET scan. Mechanical allodynia was examined using the von Frey test one day before the FDG PET scan in all SNL and sham-operated rats. FDG was intravenously injected into the tail vein via an indwelling catheter under free-moving conditions. Immediately after the FDG injection, mechanical stimulation with von Frey filaments lower than PWT in the sham-operated rats to evoke allodynia was initiated in SNL rats.

Twenty-two male Wistar rats (SLC, Hamamatsu, Shizuoka, Japan) of approximately 8 weeks of age were used for the FDG PET scan. The rats were housed in a 12-h light/dark cycle at a temperature of 22 ± 1 °C and received food and water *ad libitum*. As shown in Figure 1, the neuropathic pain model was generated by tight ligation of the left L5 and L6 spinal nerves 1-week before the FDG PET scan [19]. The rats were anesthetized with a mixture of 1.5% isoflurane and nitrous oxide/oxygen (7:3). A dorsal midline incision was made at the back from L3 to S2. The left L5 and L6 spinal nerves were isolated adjacent to the vertebral column and tightly ligated with 3-0 silk sutures. In contrast, the sham-operated control rats underwent the same surgical procedure; however, the L5/L6 spinal nerves were not ligated. The incision was then closed in two layers. The rats in the SNL group underwent a PET scan only when the paw withdrawal threshold (PWT) was less than 6.0 g in response to von Frey filaments stimulation.

### 2.2. Behavior Test

Mechanical allodynia was tested using a series of von Frey filaments applied to the plantar of the left hind paw in a blinded fashion, as described previously [34]. The rats were placed in a plastic cage with a wire mesh bottom which allowed easy access to the paws. The center-plantar surface of the left hind paw was stimulated with a series of calibrated von Frey filaments with ascending stiffness (4, 6, 8, 10, 15, and 26 g) in an incremental order starting with the lowest filament weight (4 g) after a sufficient acclimation period. Stimuli were presented at intervals of at least 30 s. A positive response was noted if the paw was removed from the wire mesh bottom. The cut-off of 26 g filament (approximately 10% of the body weight of the rats) was selected as the upper limit for testing. When the hind paw was withdrawn from a certain filament in more than two of three applications, the value of that filament in grams was considered as the PWT.

### 2.3. PET Scanning

All PET scans were performed using microPET Focus220 (Siemens Co., Ltd., Knoxville, TN, USA), which was designed for the high-resolution imaging of laboratory small animals, as previously described [35]. As shown in Figure 1, seven days after the SNL surgery, each rat underwent tail vein cannulation under anesthesia with a mixture of 1.5% isoflurane and nitrous oxide/oxygen (7:3) before the PET scan. After more than an hour of recovery, the free-moving rat received an intravenous injection of ^18^F-FDG (ca. 75 MBq/0.4 mL) in the home cage. Immediately after the FDG injection, the SNL rats were placed in the plastic cage and the left hind paw was stimulated every 30 s for 20 min with the von Frey filaments two levels higher than the corresponding PWT. The paw withdrawal rate was calculated as a percentage of positive response to total von Frey stimulations. The sham-operated control rats were kept in the home cage after the FDG injection. After a 45-min uptake period, the rats were anesthetized with a mixture of 1.5% isoflurane and nitrous oxide/oxygen (7:3), and positioned in the gantry of a PET scanner. Fifty-five minutes after receiving the ^18^F-FDG injection, a 30-min emission scan was performed with 400–650 keV as the energy window and 6 nsec as the coincidence time window. A thermocouple probe was inserted into the rectum to monitor the rectal temperature. The body temperature was maintained at approximately 37 °C with a heating blanket during the PET scan. The emission data were acquired in the list mode. The acquired data were sorted into a single sinogram. The data were reconstructed by standard 2D filtered back projection (FBP) with a Ramp filter and cutoff frequency of 0.5 cycles per pixel, or by a statistical maximum a posteriori probability algorithm (MAP), 12 iterations with point spread function (PSF) effect.

### 2.4. Image Analysis

For voxel-based statistical analysis, individual MAP-reconstructed FDG images were coregistered to an FDG template image using a mutual information algorithm with Powell’s convergence optimization method implemented with PMOD software package (version 3.2, PMOD Technologies, Ltd., Zurich, Switzerland). Subsequently, the FDG template was transformed into the space of an MRI reference template, which was placed in the Paxinos and Watson stereotactic space. The transformation parameters obtained from individual MAP-reconstructed FDG images were applied to each FBP image. For matching the default setting in SPM, the voxel size of the template was scaled by a factor of 10. Since the Paxinos stereotactic space had a slice thickness of 0.12 mm, the final voxel size was resampled at 1.2 × 1.2 × 1.2 mm. To enhance the statistical power, each FBP image was spatially smoothed with an isotropic Gaussian kernel (6-mm full width at half of the maximum [FWHM]).

The voxel-based statistical analysis was assessed using SPM8 software (Welcome Department of Imaging Neuroscience, London, UK). Proportional scaling was used for global normalization. A two-sample *t*-test was used to detect statistical differences between the treatment groups. The statistical threshold was set at *p* < 0.002 (uncorrected) with an extent threshold of 100 contiguous voxels. T-value maps of results were overlaid on the MRI template to define the voxels with significance. 

### 2.5. Data Analyses

Statistical analysis of the behavioral test was performed using GraphPad Prism 5.0 (GraphPad, San Diego, CA, USA). The statistical difference between the SNL and sham-operated rats was estimated using two-tailed unpaired *t*-tests. *p* < 0.05 was considered statistically significant. Data in the text is expressed as the mean ± standard deviation of the mean (SD).

## 3. Results

### 3.1. Mechanical Allodynia in SNL Rats

**Figure 2:**
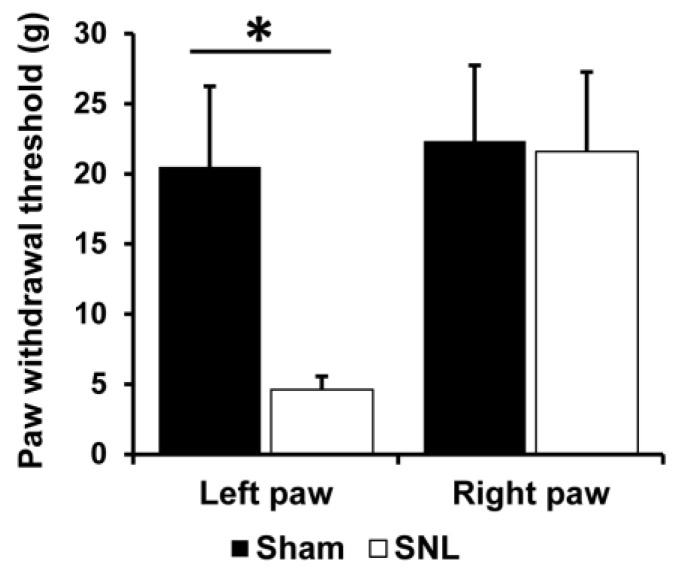
Behavior test for the assessment of mechanical allodynia one day before the FDG PET scan. Bilateral hind paw withdrawal thresholds (PWT) for von Frey filaments stimulation were assessed in the sham-operated (closed bar, *n* = 12) and SNL (open bar, *n* = 10) rats. The data are presented as mean ± standard deviation of the mean (SD). *, *p* < 0.05, two-tail unpaired *t*-tests.

SNL is one of the popular neuropathic pain models in rodents that evoked mechanical allodynia restricted to the ipsilateral hind paw for at least 2 weeks [19]. To examine mechanical allodynia, the von Frey behavior test was performed in the SNL (*n* = 10) and sham-operated groups (*n* = 12) one day before the PET scan. As shown in Figure 2, the PWT in the ipsilateral hind paw (left hind paw) was significantly decreased in the SNL group compared with that in the sham-operated group (*p* < 0.05, two-tail unpaired *t*-test). In contrast, the PWT in the contralateral hind paw of SNL rats did not show any significant change. These results indicate that mechanical allodynia was developed in the ipsilateral hind paw but not in the contralateral hind paw of SNL rats approximately one week after SNL treatment.

### 3.2. Regional Brain Activity in Response to Mechanical Stimulation

**Figure 3:**
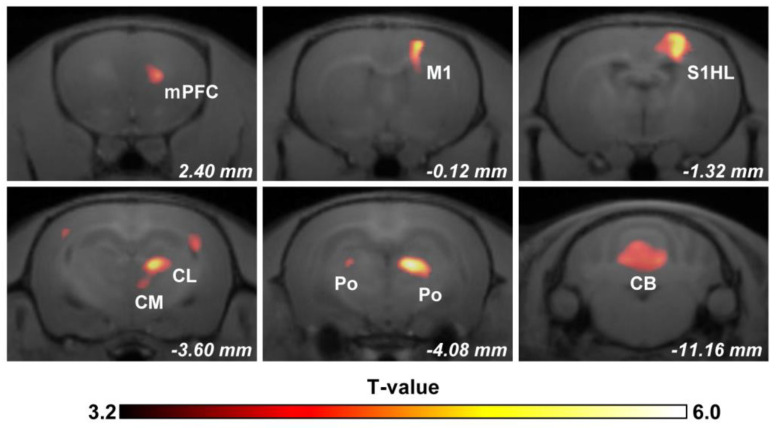
Regions of the brain activated in response to mechanical allodynia superimposed upon MRI coronal images. The images were obtained via a voxel-based statistical comparison between the FDG uptake of the SNL (*n* = 10) and sham-operated rats (*n* = 12). The T-value of 3.2 used as the threshold in the figure corresponds to the *p* < 0.002 (uncorrected) threshold. The right side of the images corresponds to the right hemisphere. The numbers in white indicate the anterior-posterior level of the coronal slices according to the rat brain atlas (Paxinos and Watson). Abbreviations: mPFC, medial prefrontal cortex; M1, primary motor cortex; S1HL, primary somatosensory cortex, hindlimb region; CL, centrolateral thalamic nucleus; CM, central medial thalamic nucleus; Po, posterior thalamic nuclei; CB, cerebellum.

To identify mechanical allodynia-related brain activity, we performed an FDG PET scan in SNL rats, wherein the rats underwent stimulation with von Frey filaments two levels higher than their own PWT (Figure 1). All von Frey filaments used for mechanical stimulation were lower than the mean value of PWT (20.5 ± 5.7 g) in the sham-operated group, indicating that von Frey stimulation was innocuous under normal conditions. Subsequently, the FDG uptake in the entire brain of these rats (*n* = 10) was compared with that of the sham-operated rats (*n* = 12) using voxel-based statistical analysis. As shown in Figure 3 and Table 1, significant activation was observed in response to mechanical allodynia in widespread regions of the brain. The regional brain activity was increased in the contralateral medial prefrontal cortex (mPFC), the primary motor cortex (M1), and the primary somatosensory cortex hindlimb region (S1HL). In the thalamus, brain activity was increased in the contralateral intralaminar nuclei, which are reported to be involved in the emotional affective component of pain, such as the centrolateral thalamic nucleus (CL) and central medial thalamic nucleus (CM). Bilateral posterior thalamic nuclei (Po) also showed significant activation, although predominant activation was observed in the contralateral hemisphere. Moreover, a vast area of the cerebellum was also activated by mechanical allodynia in SNL rats.

### 3.3. Regional Brain Activity and Behavior Correlation

**Figure 4:**
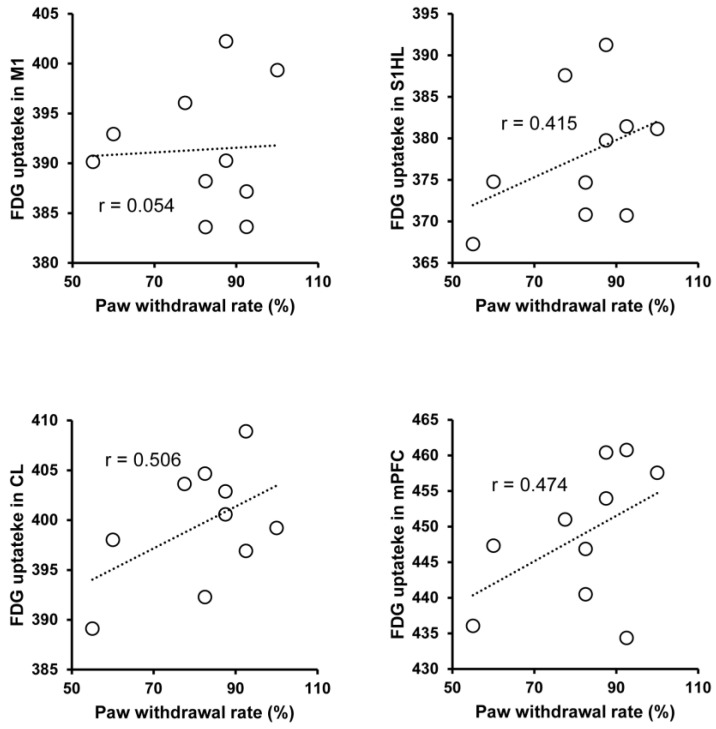
Correlation between mechanical allodynia-related behavioral changes and regional brain activity in neuropathic pain rats. The mean value of FDG uptake in the M1, S1HL CL, and mPFC were plotted against mechanical allodynia-related behavioral changes in SNL rats (*n* = 10). The Pearson coefficient value (r) is shown for each relation. Abbreviations: M1, primary motor cortex; S1HL, primary somatosensory cortex, hindlimb region; CL, centrolateral thalamic nucleus; mPFC, medial prefrontal cortex.

Finally, we further analyzed the correlation between mechanical allodynia-induced behavioral change and regional brain activity in SNL rats (*n* = 10). The calculated paw withdrawal rate in response to von Frey filaments stimulation during FDG uptake ranged from 55–100%. In these rats, the mean value of FDG uptake in the mPFC, S1HL, and CL showed a weak positive correlation with the paw withdrawal rate measured during the FDG uptake period (Figure 4). However, the mean value of FDG uptake in the M1 did not show an apparent correlation with the paw withdrawal rate.

## 4. Discussion

In the present study, we successfully identified mechanical allodynia-related brain activity in the neuropathic pain model of rats using FDG PET imaging-based small animal neuroimaging. We found that the brain activity in the pain-related regions, such as the mPFC, S1HL, CL, Po, etc. was increased in response to mechanical allodynia (Figure 3 and Table 1). Moreover, the brain activity in the high-order prefrontal cortex (mPFC), the primary somatosensory cortex (S1HL), and the intralaminar thalamic nucleus (CL) were positively correlated with mechanical allodynia-related behavioral changes, which indicated that the brain activity in these areas may encode multidimensional pain aspects. These results suggest that FDG PET imaging in conscious neuropathic pain model rats acts as a reliable biomarker for the objective assessment of pain in the preclinical study, which may bridge the inconsistencies between preclinical and clinical investigations. 

Neuroimaging has been used extensively to understand the neuronal basis of pain processing and perception, including the characterization of brain activity underlying the different dimensions of pain. The sensory-discriminative dimension of pain is thought to involve the lateral pain system, such as the lateral thalamus, sensory cortex, and posterior insular cortex [5,7,12]. Neuroimaging studies in humans and animal models have reported that the primary somatosensory cortex was activated in response to peripheral nociceptive stimulation [36]. Consistently, we demonstrated that brain activity in the contralateral S1HL, a primary somatosensory field of the hind limb, was significantly increased and positively correlated with mechanical allodynia-related behavior changes in SNL rats. Our results demonstrate that the brain activity in the S1HL could encode pain intensity and localization following neuropathic injury (Figure 3 and Figure 4). In contrast, the medial pain system is known to be involved in the emotional affective dimension of pain, such as the medial thalamus and anterior cingulate cortex [1,13,14]. In the present study, we also found significant activation in the intralaminar thalamic nuclei, such as the CL, indicating that the brain activity in the CL may be used for assessing the affective aspect of pain in neuropathic injury. Indeed, a previous lesion study further supports the engagement of the intralaminar nuclei in the pathophysiology of neuropathic pain [37]. In the present study, we also found that brain activity in the mPFC was increased and correlated with mechanical allodynia-related pain behavior. The involvement of the prefrontal cortex in different types of neuropathic pain has been reported in several clinical neuroimaging studies [8,38]. Traditionally, activation of the prefrontal cortex is thought to be related to a more cognitive evaluation dimension of pain [15,16]. Meanwhile, the frontal cortex may also be engaged in pain modulation by innervating the descending pain modulation system in the diencephalon or brainstem [39,40]. Recently, our FDG PET imaging study in neuropathic pain model rats showed that the mPFC critically contributes to pharmacological conditioning-induced placebo analgesia by interacting with the ventrolateral periaqueductal gray matter [32]. The aberrant activation of the parvalbumin interneuron in the mPFC has been found in the neuropathic animal model, and optogenetic suppression of the parvalbumin interneuron activity alleviates mechanical allodynia of neuropathic pain [39,41]. Interestingly, in brachial plexus avulsion injury model rats, the metabolic connectivity between the mPFC and several regions of the brain, such as the frontal association cortex, medial hypothalamus, diagonal band, anterodorsal hippocampus, and caudate putamen, was increased [42]. However, the pathophysiology of brachial plexus avulsion injury is complicated and involves diverse symptoms. Therefore, the pathophysiological role of the mPFC in brachial plexus avulsion injury needs to be confirmed via neurophysiological experiments in the future. Taken together, these observations suggest that the regional brain activity identified by the present FDG PET imaging study in conscious rats could be a reliable biomarker for the objective assessment of neuropathic pain in preclinical investigations.

In the present study, mechanical allodynia-related brain activity was also observed in the contralateral M1 region. Altered M1 functions have been reported in diverse pain conditions. The corticospinal output from the M1 was decreased in acute muscle pain, which may represent adaptive protection against further injury [43], whereas increased excitability of the M1 was observed in sustained muscle pain [44]. Changes in the structure, organization, and function of the M1 have been reported heterogeneously in chronic neuropathic pain [45]. M1 activation was increased in postherpetic neuralgia pain [46], and M1 cortical thickness was increased in trigeminal neuralgia pain [47]. The absence of changes in M1 activation/connectivity [48] and decreased functional connectivity in the M1 and supplementary motor cortex [49] were also reported in lower back pain. These observations suggest that the pathophysiological role of the M1 in neuropathic pain is complex and may depend largely on the pain mechanism, severity, and duration from the onset. In the present study, the regional brain activity in the M1 increased but was not correlated with paw withdrawal behavior (Figure 3 and Figure 4), indicating that the brain activity in the M1 may not encode pain intensity, at least in the current experimental setting. On the other hand, paw withdrawal behavior is a simple avoidance reflex thought to be innervated by the spinal cord, and not by the high-order motor cortex, including the M1 [22]. Therefore, the precise measurement of leg movements, such as velocity, distance, and coordinated movement, may be needed for the assessment of the functional change of the M1 of the SNL rats in the future.

The pathophysiology underlying chronic pain has been widely investigated in preclinical studies using various animal models, since mechanistic exploration using molecular, cellular, and genetic manipulation is feasible in these animal studies [17,18,19,20]. In drug development, the pharmacological efficacy of any candidate analgesic drug is primary proofed in preclinical animal models mimicking certain forms of chronic pain. However, most candidate compounds with promising efficacy in preclinical studies have failed to translate into clinical therapies [50]. This could be due to the lack of consistent biomarkers for the objective assessment of pain throughout preclinical studies to clinical application. Since pain is a subjective experience, a self-reporting-based subjective assessment is generally used as the gold standard for clinical diagnosis [3,23,24]. Whereas reflex-based behavior tests have been used widely in preclinical studies for the objective assessment of pain, such as paw withdrawal or tail-flick behavior, which are considered to measure the functional alteration in the spinal cord and brainstem but do not estimate the high-order neuropsychological processing [22]. As a potential consistent biomarker between preclinical and clinical investigations, neuroimaging has been used to identify pain-related brain activity in various animal models, such as migraine [35], neuropathic pain [27,29,51], inflammatory bowel disease [52], brachial plexus avulsion injury [42,53], and fibromyalgia [54]. In line with this, we identified hierarchical regions of the brain activated in response to mechanical allodynia in neuropathic pain model rats that are closely similar to the pain matrix defined in the human neuroimaging studies in the present study. These observations suggest that FDG PET imaging in rodents could provide a comparable objective biomarker for the consistent evaluation of pain in small animals and humans that may accelerate translational research from the preclinical to the clinical stage and increase the success rate of the development of new therapeutic drugs. Moreover, since similar regions of the brain can be identified in animal studies, molecular/cellular mechanisms of the complex signature of pain can be elucidated in animals using modern neurophysiological approaches, such as genetic manipulation. 

A major limitation of neuroimaging studies in preclinical animal models is the requirement of immobilization of the animals while scanning. In general, neuroimaging studies in laboratory animals requires the restricting of the head of the animal with anesthesia or a specific head-fix system that induces a reduction of neuronal activity [55,56]. Pain is a subjective experience where consciousness is essential for its processing. A previous neuroimaging study on neuropathic pain model rats has demonstrated that the pain-evoked activation in the somatosensory region was eliminated by anesthesia [51]. Recently, an alternative neuroimaging method based on the FDG PET scan has been widely used in small animals to avoid anesthesia and restraint stress [29,30,31,32]. In this FDG PET imaging procedure, FDG is injected under free-moving conditions and the animal can be housed in the home cage or engage in behavior tests during a certain uptake period. Subsequently, an FDG PET scan is performed under anesthesia. Since FDG is taken up by the active regions of the brain and remains within the regions for at least an hour [33], the accumulated FDG could reflect brain activity during the uptake period under conscious conditions before the PET scan. Using this alternative FDG PET imaging method, we successfully identified mechanical allodynia-related brain activity in several representative pain-related regions of the brain in neuropathic pain rats and found that brain activity in these brain regions may encode multidimensional pain aspects. Hence, the FDG PET imaging method used in the present study enabled the evaluation of pain-related brain activity without anesthesia, which might be crucial for evaluating pain processing in preclinical investigations where consciousness is necessary.

A limitation of the present study should be considered. As a representative pain assessment method, the reflex-based von Frey test was used to evaluate the pain in the SNL rats in the present study. However, such a reflex-based pain assessment is known to indicate functional alteration of the brainstem or spinal cord but not high-order neuropsychological processing [22]. This may also be a reason why the brain activity in the identified pain-related regions showed a weak positive correlation with allodynia-related behavioral changes in the present study (Figure 4). More specific behavioral assessment for high-order pain processing is needed in the future, such as a reward and escape-based operant test or a conditioned place preference test.

## 5. Conclusions

The development of a consistent biomarker for the objective assessment of pain in preclinical and clinical studies is urgently needed. In the present study, we successfully identified several regions of the brain activated by mechanical allodynia in neuropathic pain rats using an alternative neuroimaging method based on an FDG-PET scan. Moreover, activated brain activity in the sensory-discrimination (S1HL), cognitive evaluation (mPFC), and emotional affection (CL) regions correlated with mechanical allodynia-related behavioral changes. These results indicate that the brain activity in these areas may encode different dimensions of pain components, and current alternative FDG PET imaging procedures in rodents could be a powerful tool for providing a consistent biomarker for the objective assessment of pain throughout preclinical to clinical studies. Thus, our study will lead to the elucidation of the complex signature of pain and help in the management of patients with chronic pain.

## Figures and Tables

**Table 1:** Regions of the brain activated in response to mechanical allodynia in a neuropathic pain rat model.

Brain Regions	Laterality	T-Value (Peak)	Volume (mm^3^)
Anterior olfactory nucleus lateral part (AOL)	R	3.97	0.32
Medial prefrontal cortex (mPFC)	R	4.45	1.49
Cluster 1			15.44
Primary motor cortex (M1)	R	5.3	
Primary somatosensory cortex hindlimb region (S1HL)	R	5.39	
Primary somatosensory cortex barrel field (S1BF)/ Primary somatosensory cortex dysgranular zone (S1DZ)	L	3.89	0.85
Cluster 2			7.54
Centrolateral thalamic nucleus (CL)	R	5.35	
Central medial thalamic nucleus (CM)	R	3.81	
Posterior thalamic nucleus (Po)	R	6.55	
Posterior thalamic nucleus (Po)	L	3.98	0.34
Cerebellum (CB)	R/L	5.92	30.06

SNL + stimuli (*n* = 10) vs. sham-operated (*n* = 12). Height threshold: T = 3.25, *p* < 0.002 (uncorrected). R and L indicate right and left side hemispheres, respectively.

## Data Availability

The data that support the findings of this study are available from the corresponding author upon reasonable request.
